# Milk proteome from in silico data aggregation allows the identification of putative biomarkers of negative energy balance in dairy cows

**DOI:** 10.1038/s41598-019-46142-7

**Published:** 2019-07-04

**Authors:** Mylène Delosière, José Pires, Laurence Bernard, Isabelle Cassar-Malek, Muriel Bonnet

**Affiliations:** Université Clermont Auvergne, INRA, VetAgro Sup, UMR Herbivores, F-63122 Saint-Genès-Champanelle, France

**Keywords:** Gene ontology, Data integration, Animal physiology

## Abstract

A better knowledge of the bovine milk proteome and its main drivers is a prerequisite for the modulation of bioactive proteins in milk for human nutrition, as well as for the discovery of biomarkers that are useful in husbandry and veterinary medicine. Milk composition is affected by lactation stage and reflects, in part, the energy balance of dairy cows. We aggregated the cow milk proteins reported in 20 recent proteomics publications to produce an atlas of 4654 unique proteins. A multistep assessment was applied to the milk proteome datasets according to lactation stages and milk fractions, including annotations, pathway analysis and literature mining. Fifty-nine proteins were exclusively detected in milk from early lactation. Among them, we propose six milk proteins as putative biomarkers of negative energy balance based on their implication in metabolic adaptative pathways. These proteins are PCK2, which is a gluconeogenic enzyme; ACAT1 and IVD, which are involved in ketone metabolism; SDHA and UQCRC1, which are related to mitochondrial oxidative metabolism; and LRRC59, which is linked to mammary gland cell proliferation. The cellular origin of these proteins warrants more in-depth research but may constitute part of a molecular signature for metabolic adaptations typical of early lactation.

## Introduction

Phenotyping animal traits related to performance, quality, welfare and health is often challenging but necessary to meet husbandry and societal expectations. Rapid and non-invasive tools are desirable to monitor multiple animals traits accurately and inexpensively^1–3^. Biological fluids such as milk are increasingly used as a source for animal trait phenotyping^3^. Milk composition varies depending on several factors, including the stage of lactation, the metabolic status and the health status of dairy cows^3^. In early lactation, energy balance and body reserve mobilization are major drivers of dairy performance, robustness and longevity of dairy cows^4^. Negative energy balance (**NEB**) occurs frequently in early lactation cows because the energy demands for milk production exceed nutrient intake^5–7^. Maladaptation during the periparturient period impacts milk production and increases the risk of post-partum diseases, removal from the herd and infertility^8,9^. Much attention has been paid to the development of milk indicators for NEB in high-producing dairy cows. Of these, some are based on proteins identified by proteomics. The abundance of ten proteins with roles in cholesterol synthesis and composition of the milk fat globule membrane (MFGM) was shown to vary with the energy balance in early lactation cows^10^. Furthermore, improvements in the sensitivity of proteomics techniques^11^, which have allowed the identification of thousands of proteins in milk^12,13^; have contributed to a better understanding of lactation periods^10,14^ and animal health^15–17^. The volume of publicly available proteomics data provides opportunities for in silico proteomics studies in compliance with recent guidelines^18^. We hypothesized that the computation of available proteomics data would allow putative biomarkers of NEB to be identified. We used the early lactation period as a proxy for NEB because modern dairy cows systematically experience some degree of negative energy balance and extensive mobilisation of body reserves during this period. We use, reuse, reprocess, and repurpose^18^ the cow milk proteome reported in 20 recent publications to describe the proteomic signature of milk according to lactation period and milk fraction. The aim of the current study was to identify proteins that were specifically identified in early lactation milk. These proteins could be robust biomarkers of NEB based on their presence or absence in milk or milk fractions during early lactation (all or nothing identification). Moreover, because available proteomics data were obtained from different breeds, countries and rearing practices, these proteins may constitute robust biomarkers of NEB, independently of the breed and husbandry practices. To our knowledge, this is the first attempt to reuse the publicly available milk proteome data to propose potential indicators for NEB and for dairy ruminants.

## Results

### Proteome atlas overview according to milk fractions

**Figure 1:**
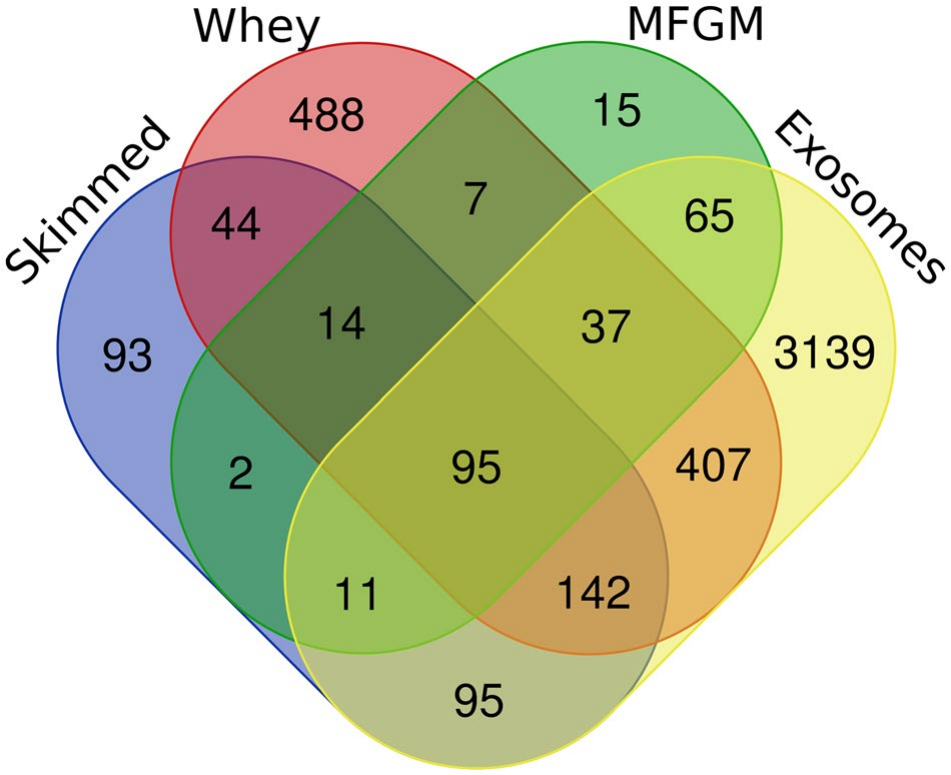
Venn diagram of common and specific gene names for proteins present in skimmed, whey, MFGM and exosome milk fractions.

Among the 4654 proteins compiled in the atlas, 95 gene names (**GN**) (Fig. 1 and Supplementary information Table 1) were detected for all four milk fractions, whereas 93, 488, 15 and 3139 GN were specifically detected in skimmed milk, whey, MFGM and exosomes.

The 95 GN detected in the studied milk fractions are the most abundant milk proteins. These included alpha-S1-casein (**CSN1S1**), alpha-S2-casein (**CSN1S2**), beta-casein (**CSN2**), α-lactalbumin (**LALBA**) and β-lactoglobulin (**LGB**), with the latter frequently detected in whey. The 8 major proteins detected in the MFGM, such as mucin 1 (**MUC1**), redox enzyme xanthine dehydrogenase (**XDH**), and butyrophilin subfamily 1 member A1 (**BTN1A1**), were among the list of 95 GN. Similarly, proteins classically detected in milk exosomes, such as lipopolysaccharide-binding protein (**LBP**), annexin (**ANXA1**), complement c3 (**C3**), protein S100-A9 (**S100A9**), serum amyloid A protein (**SAA3**) and cathelicidin-1 (**CATHL1**), were also among the list of 95 proteins found in all the milk fractions. Enriched Gene Ontology (**GO**) terms (980 GO terms) annotated 94 proteins among the 95 detected proteins in the four milk fractions. Among the top 50 enriched (P < 0.05) GO terms in the Biological Process (BP) category, 9 terms related to lipoprotein, phosphatidylcholine and cholesterol, highlighting the proteins involved in lipid metabolism. Other enriched GO terms were related to hormone and cytokine signalling, such as the growth hormone secretion that annotated a member of the RAS oncogene family protein (**RAB1A**). The growth factor/hormone term annotated the heat shock protein HSP 90-beta (**HSP90AB1**) and **CSN1S1**. Terms linked to inflammatory response annotated the tissue factor (F3) involved in the cytokine-mediated signalling pathway and the proteins S100A8 and S100A9 that are involved in antioxidant activity and immune response.

## Proteome atlas overview according to lactation stages

**Figure 2:**
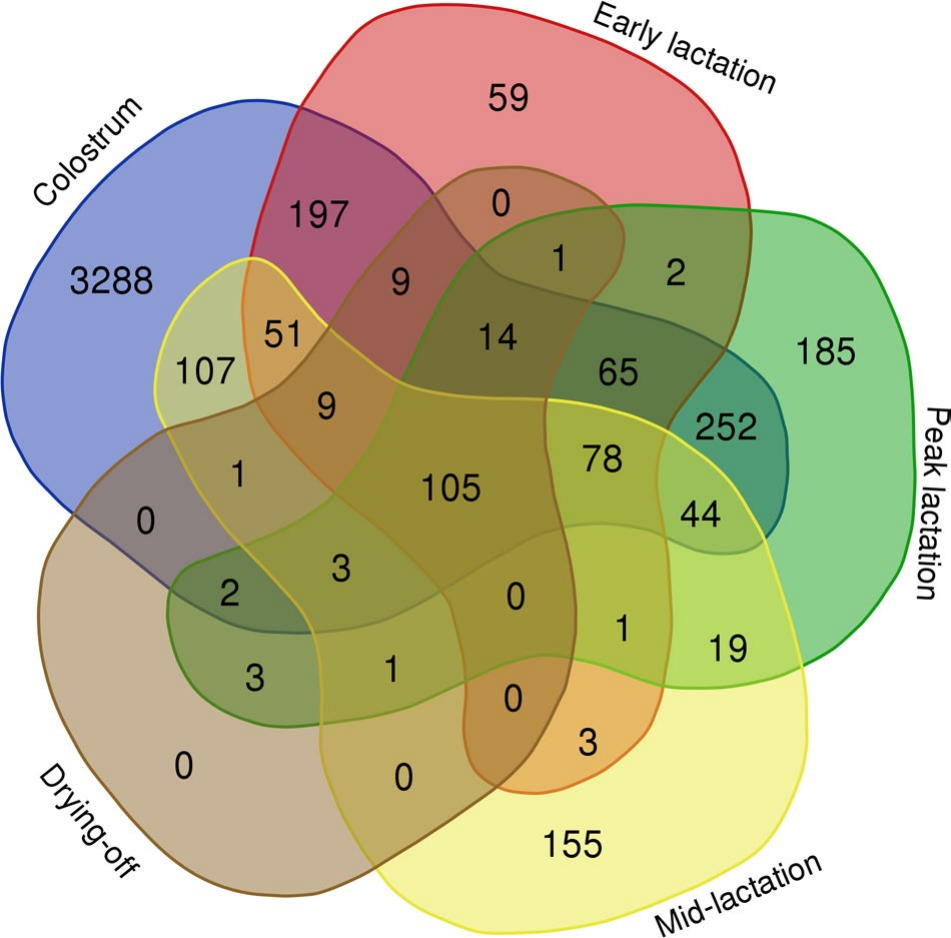
Venn diagram of common and specific gene names from the colostrum period, early lactation, peak lactation, mid-lactation and drying-off.

Of the 4654 proteins present in the atlas, 105 GN (Fig. 2 and Supplementary information Table 1) were detected in all five lactation stages, whereas 3288, 59, 185 and 155 GN were exclusively identified during the colostrum period and early, peak and mid-lactation.

As expected, of the 105 GN, four were the major caseins: CSN1S1, CSN1S2, CSN2 and kappa-casein (**CSN3**). Other detected proteins related to protein and lactose synthesis, e.g., lactotransferrin (**LTF**), LALBA and beta-1,4-galactosyltransferase 1 (**B4GALT1**). Some proteins linked to lipolysis and fatty acid esterification were also detected, including perilipin-2 (**PLIN2**); apolipoproteins E, A-IV, A-I (**APOE, APOA4, APOA1**); and lipoprotein lipase (**LPL**), which in the mammary gland hydrolyses the triglycerides circulating in chylomicra and very low density lipoproteins, and in milk hydrolyses the triglycerides of the fat globule core. F 45 kDa calcium-binding protein (**SDF4**), which is linked to calcium metabolism, and leucine-rich alpha-2-glycoprotein 1 (**LRG1**), implicated in brown fat cell differentiation, were also detected. Six hundred seventy-eight enriched GO terms annotated 101 of the 105 proteins detected in all lactation stages. Among the top 50 (P < 0.05) of the enriched GO terms in the BP category, one term related to phosphatidylcholine annotated heart fatty acid-binding protein (**FABP3**) and highlighted lipid metabolism. The term lactose biosynthetic process and 3 terms related to hormones annotated LALBA, B4GALT1 and CSN1S1, which are all proteins that support lactation.

### Focus on milk proteins specific for early lactation as potential biomarkers of NEB

The 59 GN exclusively detected during early lactation (i.e., 6 to 21 days in milk (**DIM**); Fig. 2) are listed in Table 1.

**Table 1:** The 59 proteins detected in milk during early lactation (i.e., 6 to 21 DIM).

No.	Gene Name	Protein ID	Protein name	Milk fraction localization	References
1	ABCC4	A0JND8_BOVIN	ABCC4 protein	MFGM	^10^
2	ACAT1	THIL_BOVIN	Acetyl-CoA acetyltransferase, mitochondrial	Skimmed	^13, 17^
3	ALDH3B1	AL3B1_BOVIN	Aldehyde dehydrogenase family 3 member B1	Skimmed	^10^
4	ALOX12	Q6SYC4_BOVIN	Arachidonate 12-lipoxygenase	MFGM	^10^
5	ARL6IP5	PRAF3_BOVIN	PRA1 family protein 3	Skimmed	^13, 17^
6	ATP5F1^a^	AT5F1_BOVIN	ATP synthase F(0) complex subunit B1, mitochondrial	Skimmed	^13, 17^
7	ATP5H^a^	ATP5H_BOVIN	ATP synthase subunit d, mitochondrial	Skimmed	^13, 17^
8	ATP5J^a^	ATP5J_BOVIN	ATP synthase-coupling factor 6, mitochondrial	Skimmed	^13, 17^
9	ATP5J2^a^	ATPK_BOVIN	ATP synthase subunit f, mitochondrial	Skimmed	^13, 17^
10	BAT1	Q861P7_BOVIN	HLA-B-associated transcript 1	Skimmed	^13^
11	Bt.64131	F1MIR4	RAB2A, member RAS oncogene family	MFGM	^10^
12	C13H20ORF116^a^	Q1LZB0_BOVIN	Chromosome 20 open reading frame 116 ortholog	Skimmed	^13^
13	C1QBP	C1QBP_BOVIN	Complement component 1 Q subcomponent-binding protein, mitochondrial	Skimmed	^13, 17^
14	CAT	CATA_BOVIN	Catalase	Skimmed	^13, 17^
15	COPG	Q0V888_BOVIN	Coatomer protein complex, subunit gamma 1	Skimmed	^13, 17^
16	COX5A	COX5A_BOVIN	Cytochrome c oxidase subunit 5A, mitochondrial	Skimmed	^13, 17^
17	COX5B	COX5B_BOVIN	Cytochrome c oxidase subunit 5B, mitochondrial	Skimmed	^13, 17^
18	COX7A2	CX7A2_BOVIN	Cytochrome c oxidase subunit 7A2, mitochondrial	Skimmed	^13, 17^
19	CUZD1^a^	F1MD73	Uncharacterized protein	MFGM	^10^
20	DDRGK1	DDRGK_BOVIN	DDRGK domain-containing protein 1	Skimmed	^13, 17^
21	DDX39B	DX39B_BOVIN	Spliceosome RNA helicase DDX39B	Skimmed	^17^
22	EEF1B	EF1B_BOVIN	Elongation factor 1-beta	Skimmed	^13, 17^
23	GPAM	GPAT1_BOVIN	Glycerol-3-phosphate acyltransferase 1, mitochondrial	MFGM	^10^
24	HIST1H4A^a^	P62803	Histone 4	MFGM	^10^
25	IG^a^	A5D7Q2	Uncharacterized protein	MFGM	^10^
26	IVD	IVD_BOVIN	Isovaleryl-CoA dehydrogenase, mitochondrial	Skimmed	^13, 17^
27	LF	Q95M55_BOVIN	Lactoferrin	MFGM	^10^
28	LMAN1	Q8MJ82_BOVIN	Lectin mannose binding 1	Skimmed	^13, 17^
29	LOC789567	A6H7H3_BOVIN	LOC789567 protein	Skimmed	^13, 17^
30	LRPAP1	Q148K7_BOVIN	Low density lipoprotein receptor-related protein associated protein 1	Skimmed	^13, 17^
31	LRRC59	LRC59_BOVIN	Leucine-rich repeat-containing protein 59	Skimmed	^13, 17^
32	Man8	O78186_BOVIN	MHC class I antigen	MFGM	^10^
33	MGC137099	Q2KII3_BOVIN	Hepatitis A virus cellular receptor 1 N-terminal domain containing protein	MFGM	^10^
34	MYCBP	MYCBP_BOVIN	c-Myc-binding protein	Skimmed	^13, 17^
35	NDUFA5	NDUA5_BOVIN	NADH dehydrogenase [ubiquinone] 1 alpha subcomplex subunit 5	Skimmed	^13, 17^
36	NDUFAB1	ACPM_BOVIN	Acyl carrier protein, mitochondrial	Skimmed	^13, 17^
37	OSTC	OSTC_BOVIN	Oligosaccharyltransferase complex subunit OSTC	Skimmed	^13^
38	PAFAH1B2	PA1B2_BOVIN	Platelet-activating factor acetylhydrolase IB subunit beta	Skimmed	^13, 17^
39	PCK2	F1MDS3_BOVIN	Phosphoenolpyruvate carboxykinase 2, mitochondrial	Skimmed	^13, 17^
40	PLSCR2	PLS2_BOVIN	Phospholipid scramblase 2	MFGM	^10^
41	RPL10A	RL10A_BOVIN	60S ribosomal protein L10a	Skimmed	^13, 17^
42	RPL18	RL18_BOVIN	60S ribosomal protein L18	Skimmed, MFGM	^10, 13, 17^
43	RPL6	RL6_BOVIN	60S ribosomal protein L6	Skimmed	^13, 17^
44	RPL7	RL7_BOVIN	60S ribosomal protein L7	Skimmed	^13, 17^
45	RPL7A	RL7A_BOVIN	60S ribosomal protein L7a	Skimmed	^13, 17^
46	RPS13	RS13_BOVIN	40S ribosomal protein S13	Skimmed	^13, 17^
47	SAA	P35541	Serum amyloid A protein	MFGM	^10^
48	SDHA	SDHA_BOVIN	Succinate dehydrogenase [ubiquinone] flavoprotein subunit, mitochondrial	Skimmed	^13, 17^
49	SEC. 11C	Q2KI36_BOVIN	Signal peptidase complex catalytic subunit SEC. 11	Skimmed	^13, 17^
50	SEC. 61A1	S61A1_BOVIN	Protein transport protein Sec. 61 subunit alpha isoform 1	Skimmed	^13, 17^
51	SLC15A2	B8Y6C2_BOVIN	Solute carrier family 15 member 2	MFGM	^10^
52	SSR1	SSRA_BOVIN	Translocon-associated protein subunit alpha	Skimmed	^13, 17^
53	SURF4	SURF4_BOVIN	Surfeit locus protein 4	Skimmed	^13, 17^
54	SYPL1	A8PVV5_BOVIN	SYPL1 protein	Skimmed	^13, 17^
55	TMED9	TMED9_BOVIN	Transmembrane emp24 domain-containing protein 9	Skimmed	^13, 17^
56	TMEM43	A6QQR5_BOVIN	TMEM43 protein	Skimmed	^13, 17^
57	TREM1	TREM1_BOVIN	Triggering receptor expressed on myeloid cells 1	Skimmed	^13, 17^
58	TXNDC4^a^	TXND4_BOVIN	Thioredoxin domain-containing protein 4	Skimmed	^13^
59	UQCRC1	QCR1_BOVIN	Cytochrome b-c1 complex subunit 1, mitochondrial	Skimmed	^13, 17^

^a^Updated since the period of data mining. The Gene name and ID were converted before November 2018 by use of the Retrieve/ID Mapping tool of the Uniprot database.

Of the 59 GN, 52 were annotated by 326 enriched GO terms. Among the most significantly (P < 0.001) enriched GO terms in the BP category (Supplementary information Fig. 1), 3 terms are related to the oxidation-reduction process and oxidative metabolism. These terms annotated proteins of the respiratory chain such as the mitochondrial cytochrome b-c1 complex subunit 1 protein (**UQCRC1**); mitochondrial acyl carrier protein (**NDUFAB1**); and two mitochondrial cytochrome c oxidase subunits, 5B and 5A (**COX5B, COX5A**), which are terminal oxidases in mitochondrial electron transport. We listed the mitochondrial succinate dehydrogenase flavoprotein subunit (**SDHA**), which is involved in the complex II of the mitochondrial electron transport chain. We identified the aldehyde dehydrogenase family 3 member B1 (**ALDH3B1**), which oxidizes medium- and long-chain saturated and unsaturated aldehydes, and the mitochondrial isovaleryl-CoA dehydrogenase (**IVD**) that is involved in the synthesis of 3-hydroxy-3-methylglutaryl-CoA from 3-isovaleryl-CoA as it enters the β-oxidation step. The terms related to oxidative metabolism also annotated catalase (**CAT**), which protects cells from the toxic effects of hydrogen peroxide, and the 12 S type arachidonate 12-lipoxygenase (**ALOX12**), which participates in lipid hydroperoxidation. Seven terms are related to translation and transport. One term, ribosome biogenesis, annotated the mitochondrial complement component 1 Q subcomponent-binding protein (**C1QBP**). Three terms are related to ketone metabolism and annotated the mitochondrial acetyl-CoA acetyltransferase or acetoacetyl-CoA thiolase (**ACAT1**). Other milk proteins were not annotated and may contribute to the oxidative metabolism, such as the mitochondrial phosphoenolpyruvate carboxykinase 2 (**PCK2**), the mitochondrial glycerol-3-phosphate acyltransferase 1 (**GPAM**), the triggering receptor expressed on myeloid cells 1 (**TREM1**) and the signal peptidase complex catalytic subunit (**SEC. 11C**). The Leucine-rich repeat-containing protein 59 (**LRRC59**) is also annotated in early lactation milk. This protein is required for nuclear import of the fibroblast growth factor 1 (**FGF1**).

Algorithms implemented in ProteINSIDE proposed 15 out of the 59 proteins (25% of the early milk proteins list) as mitochondrial proteins. The mitochondrial proteins are COX7A2, NDUFA5, GPAM, ATP5F1, ATP5H, ATP5J, ATP5J2, C1QBP, COX5B, COX5A, NDUFAB1, SDHA, UQCRC1, IVD and ACAT1. Finally, several proteins belong to protein complexes of the ATP synthase (4 proteins), cytochrome c oxidase (3 proteins) and 60S ribosomal protein (5 proteins).

Of these 59 proteins, only the RPL18 was detected in both the MFGM and skimmed milk fractions. Fourteen proteins were detected exclusively in the MFGM fraction: SLC15A2, Man8, Bt.64131, the 12S type arachidonate 12-lipoxygenase (**ALOX12**), GPAM, HIST1H4A, PLSCR2, LF, ABCC4, CUZD1, IG, MGC137099, ALDH3B1 and SAA. The 44 other proteins were detected exclusively in the skimmed milk (Table 1). Obviously, these proteins are also present in whole milk. However, the ability to detect and quantify minor proteins will strongly depend on their concentration; therefore, their technical enrichment in these fractions before mass spectrometry identification could be valuable.

## Discussion

We merged available proteomic datasets to produce an atlas of 4654 nonredundant proteins. We identified 59 proteins specifically found in early lactation milk, which we proposed as putative biomarkers of NEB. Of these, we focused on six milk proteins because of their link with ketogenesis, gluconeogenesis and oxidative metabolism, which are well known metabolic pathways enhanced in dairy cows during early lactation.

Early lactation is a classical situation of physiological undernutrition and NEB because feed intake increases at a slower pace than the requirements for milk production. The prioritization of nutrient partitioning to the mammary gland and milk synthesis leads to mobilization of body fat, glycogen, proteins and minerals. Dairy cows may mobilize up to 90 kg of fat and 24 kg of protein^7^. Intense lipomobilisation leads to the release of adipose free fatty acids (**FFA**) into plasma. At the same time, lipogenesis, FA esterification and glucose utilization decrease in adipose tissue^7^. Much of the mobilized protein appears to be derived primarily from skeletal muscle through the downregulation of tissue protein synthesis and increased proteolysis^19^. The liver coordinates and interconverts nutrients to support pregnancy and lactation by increasing gluconeogenesis and ketogenesis^6,7^. The NEB of dairy cows leads to these metabolic adaptations during early lactation^20,21^. We mined the 59 proteins exclusively detected in milk sampled between 6 and 21 DIM from cows likely in NEB, relative to these well-known homeorhetic adaptations.

**Figure 3:**
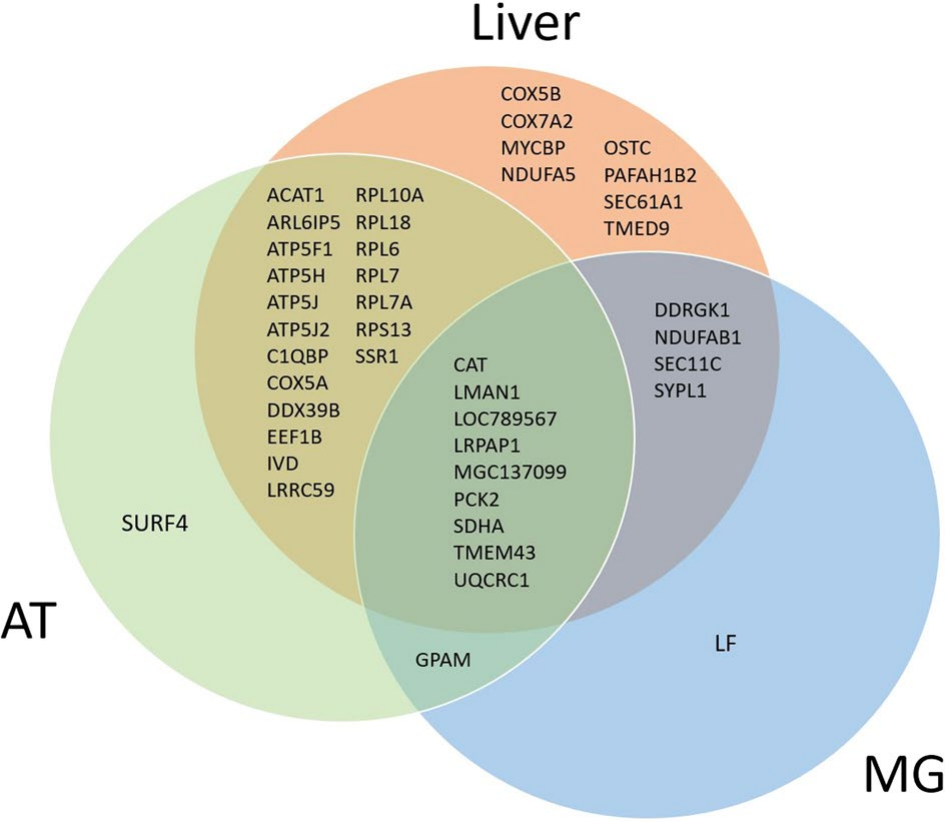
Venn diagram of the 43 gene names from early milk proteins that were previously identified in the liver^24^, adipose tissue^25^ and mammary gland^23^ of dairy cows.

All 59 proteins may be putative biomarkers of NEB. None of them were previously listed in a study reporting the variation in abundance of milk protein relatively to the energy status of dairy cows^10^ or in a comparison between colostrum and 7 DIM^22^. Despite being present in our atlas, 11 proteins (stomatin (**STOM**), ectonucleotide pyrophosphatase/phosphodiesterase family member 3 (**ENPP3**), acyl-CoA synthetase long-chain family member 1 (**ACSL1**), NADH-cytochrome b5 reductase 3 (**CYB5R3**), Isocitrate dehydrogenase [NADP] cytoplasmic (**IDH1**), lactoperoxidase (**LPO**), serum albumin (**ALB**), LGB, LALBA, LBP and cell death-inducing DFFA-like effector a (**CIDEA**)) were not specific to early lactation and therefore were not included in our present/absent list. We believed that the binary approach that we utilized has produced a robust list of biomarkers because these proteins were present exclusively in milk from cows in early lactation. This suggests that their abundance strongly increases during early lactation, which may simplify their detection and quantification to qualify these biomarkers relative to the energy status of cows in further studies. Moreover, the reliability of the 59 putative biomarkers of NEB that we propose is strengthened because most of them were previously identified as affected by lactation stage^23^ and heat stress^24,25^ during early lactation. Indeed, out of the 59 proteins detected in early lactation milk, 43 were previously identified in the adipose tissue^25^, liver^24^ or mammary gland^23^ of lactating cows experiencing different EB (Fig. 3). Of these, 12 proteins belong to 3 protein complexes (ATP synthase, cytochrome c oxidase and 60S ribosomal protein), which reduced the diversity of proteins on our list. However, for future studies aimed to evaluate these putative biomarkers of NEB in compliance with the biomarker discovery pipeline^26^, we need to focus on a restricted number of proteins. Therefore, we selected 6 proteins we believe are pertinent because of their biological functions relative to the homeorhetic adaptation of early lactation cows. These proteins, which are the mitochondrial phosphoenolpyruvate carboxykinase 2 (**PCK2**), **ACAT1**, **IVD**, **SDHA**, **UQCRC1** and **LRRC59**, are potential indicators of NEB because of their roles in metabolic adaptations to early lactation.

We propose LRRC59, a protein originating from the cytosol, membrane and/or endoplasmic reticulum, as a putative biomarker of NEB. This protein was identified in the AT and liver proteomes^24,25^, but not in mammary gland. The LRRC59 is required for the nuclear import of the FGF1^27^, a growth factor that participates in the regulation of proliferation and differentiation of the mammary gland cells. Immunoreactive FGF1 was found in considerable concentration in the epithelium of the mammary gland in heifers during mammogenesis and lactation^28^. The LRRC59 is a membrane‐anchored protein located in the endoplasmic reticulum that may be secreted in milk via exocytosis.

PCK2 is the mitochondrial isoform of the hepatic gluconeogenic enzyme that was detected in early lactation milk. The massive increase in glucose requirements around calving is partially met by endogenous glucose synthesis via liver gluconeogenesis in dairy cows. The gene expression of PCK2 was increased in the liver of cows in early lactation^29^, and the processes of milk secretion may transfer the PCK2 protein from the plasma into the milk. Indeed, in humans, the mapping of the human tissue proteome based on an integrated omics analysis has indicated that the liver is the major secretory tissue^30^. However, milk PCK2 could also arise from a mammary synthesis, since the abundance of PCK2 mRNA in the mammary tissue, as in liver and in skeletal muscle, varied depending on the genetic merit and lactation performances of the dairy cows^31^. Moreover, PCK2 activity was reported to increase 43-fold during the transition from pregnancy into lactation in the guinea pig mammary gland^32^. The biological function of PCK2 expression by the liver or other organs may be to provide phosphoenolpyruvate for gluconeogenesis. Due to the similarity between PCK2 and PCK1, PCK2 was also proposed to contribute to the synthesis of glycerol-3-phosphate, a precursor for fatty acid esterification into triglycerides, especially in tissues deprived of glucose-6-phosphatase such as mammary gland^31^.

We proposed ACAT1 as a potential biomarker of NEB because it plays a major role in ketone metabolism. Early lactating cows mobilize lipids stored in adipose tissue and exhibit an increased plasma FFA concentration. Plasma FFA are taken up by the liver and partially oxidized to ketone bodies. Milk β-hydroxybutyrate, a ketone body, is commonly used to detect subclinical ketosis^33,34^, which is consistent with the detection of ACAT1 in early lactation milk.

We proposed IVD as a potential NEB biomarker because it is also related to ketogenesis. IVD is involved in the L-leucine degradation pathway and in the synthesis of 3-hydroxy-3-methylglutaryl-CoA, an intermediate in ketogenesis. IVD was detected in bovine liver^35^, AT proteomes^25^, and in mammary gland transcriptome^36^. The origin and biological significance of milk ACAT1 and IVD remain unclear. Nevertheless, these proteins may constitute molecular signatures of a ketogenic state typical of early lactation.

We proposed SDHA and UQCRC1, two mitochondrial proteins involved in the oxidative phosphorylation and ATP production, as putative biomarkers of NEB. SDHA is involved in the complex II of the mitochondrial electron transport chain and has a role in the tricarboxylic acid cycle pathway. The SDHA was reported in milk somatic cells^37^. During lactation, mammary fatty acid and cholesterol synthesis require large amounts of energy and reduction equivalents in the form of NADPH. In the ruminant mammary gland, most NADPH is synthesised from the decarboxylation of isocitrate^5^. Therefore, the presence of SDHA in milk may reflect NADPH requirements and the oxidative metabolic activity of the mammary gland. The UQCRC1 is involved in complex III of the respiratory chain, electron transfer coupled to proton pumping and NADPH synthesis and was reported in bovine mammary gland^23,36^. Mitochondrial NADPH production implies the transfer of electrons and the translocation of protons. We speculate that UQCRC1 in milk reflects the oxidative metabolic activity of the mammary gland. The presence of these five mitochondrial proteins (PCK2, ACAT1, IVD, SDHA and UQCRC1) in early lactation milk reflects the upregulation of metabolic pathways (ketogenesis, respiratory chain, tricarboxylic acid cycle and β-oxidation cycle) in the mammary gland and other key tissues of early lactation cows experiencing NEB and a glucose deficit. The identification of several mitochondrial proteins is consistent with the increase in the number of mitochondria present in bovine epithelial cells from parturition until the peak of lactation, which emphasizes the pertinence of mitochondrial proteins as putative biomarkers of NEB^38^.

Our in silico proteomics approach allowed us to produce an atlas of milk proteins, of which we listed 59 milk proteins as being present exclusively in early lactation milk (post-colostrum period), a period characterized by NEB, lipomobilisation and metabolic imbalance in dairy cows. Among this list of putative biomarkers of NEB, we selected six proteins—LRRC59, PCK2, ACAT1, IVD, SDHA and UQCRC1—based on their roles in multiple pathways of energy metabolism, and therefore potentially associated with states of metabolic imbalance. All six proteins were detected in skimmed milk; consequently, technological enrichment of these proteins may be possible in further assays. Notably, given the power of proteomics, minor proteins can be detected despite their low concentration. Whether these proteins arise from tissues that are central in the homeorhetic adaptation of early lactation warrants further study. Further research is also needed to qualify these biomarkers when a relationship between the abundance of these proteins in milk and the energy status of early lactating cows is implied. The results obtained in this study proves the usefulness of mining the present atlas to understand and phenotype some traits of dairy cows.

## Methods

### Construction of the milk proteome atlas

A computational workflow was used to aggregate data from publications reporting cow milk proteome to create an atlas of all proteins present, independently of their abundance and without statistical analysis. Briefly, we collected publications on bovine milk by a literature search on *Bos taurus* using PubMed.gov (NCBI) and the Web of Science (Clarivate Analytics) search engines up to February 2018. The search provided 87 milk proteome publications that were reviewed and curated based on the availability of information, such as the days in milk and the health status of the cows, as well as accessible supplementary materials. Milk protein data could come from tank samples or individual milking but only from cows without mammary infection according to the somatic cell counts or as declared by the authors. Twenty publications complied with the selection criteria:^10,13,15,17,22,39–53^. The main objective of this computational data aggregation from 20 publications, with 35 datasets (Table 2), is to obtain an overview of milk proteins independently of breed, age, country and methodologies of protein isolation and identification. Methods for protein isolation were density gradient ultracentrifugation^15,39^, centrifugation and washing^10,22,40^, (ultra)centrifugation with acidification^48^ and with major proteins depletion^42,45,46^, or (ultra)centrifugation without acidification^13,17,41,43,44,47,49–53^.

**Table 2:** Characteristics of the 35 datasets selected for data extraction.

Datasets	Variation factor studied by authors	Factor selected for data extraction	Milk fraction class	Lactation stage class	Country	Breed	Sampling	Protein isolation	Protein identification^a^	Ref
1	lactation stage	12 and 24 h post-partum	Exosomes	Colostrum	Australia	Holstein-Friesian	Fresh	Density gradient ultracentrifugation	nanoLC-MS/MS	^15^
2	lactation stage	48 h post-partum	Exosomes	Colostrum	Australia	Holstein-Friesian	Fresh	Density gradient ultracentrifugation	nanoLC-MS/MS	^15^
3	lactation stage	72 h post-partum	Exosomes	Colostrum	Australia	Holstein-Friesian	Fresh	Density gradient ultracentrifugation	nanoLC-MS/MS	^15^
4	milk maturation	0 to 5 d post-partum	Exosomes	Colostrum	China	Holstein	Fresh	Density gradient ultracentrifugation	iTRAQ labelling, SCX, nano-LC-MS/MS	^39^
5	lactation stage	1 d post-partum	MFGM	Colostrum	USA	Holstein	Fresh	Centrifugation, washing	iTRAQ labelling, SCX, nano-LC-MS/MS	^22^
6	milk characterization	7 d post-partum	MFGM	Early lactation	USA	Holstein	Fresh	Centrifugation, washing	iTRAQ labelling, SCX, nano-LC-MS/MS	^22^
7	lactation stage	dry period length	MFGM	Early lactation	not reported	Holstein-Friesian	Snap-frozen (−20 °C)	Centrifugation, washing	FASP, dimethyl labelling, LC-LTQ-Orbitrap/MS	^10^
8	breed	Holstein	MFGM	Early lactation	China	Holstein	Fresh	Centrifugation, washing	LC-MS/MS	^40^
9	milk maturation	48 h post-partum	Skimmed milk	Colostrum	Brazil	Holstein	Fresh	Acidification, centrifugation, major protein depletion	2D Maldi-TOF/TOF-MS	^42^
10	milk maturation	72 h post-partum	Skimmed milk	Colostrum	Brazil	Holstein	Fresh	Acidification, centrifugation, major protein depletion	2D Maldi-TOF/TOF-MS	^42^
11	milk characterization	1 d post-partum	Skimmed milk	Colostrum	Denmark	Holstein-Friesian	Snap-frozen (−80 °C)	Centrifugation	2D LC-MS/MS	^41^
12	lactation stage	1 d post-partum	Skimmed milk	Colostrum	Denmark	Holstein-Friesian	Fresh	Centrifugation	2D LC-MS/MS	^17^
13	lactation stage	10 d post-partum	Skimmed milk	Early lactation	Denmark	Holstein-Friesian	Fresh	Centrifugation	2D LC-MS/MS	^17^
14	milk characterization	milk fractionation	Skimmed milk	Early lactation	Denmark	Holstein-Friesian	Fresh	Centrifugation	2D LC-MS/MS	^13^
15	diet	low RDP:RUP^b^ ratio	Skimmed milk	Peak lactation	USA	Holstein	Fresh, bronopol and natamycin addition, snap frozen (−80 °C)	Centrifugation, sonication	HPLC	^45^
16	diet	high RDP:RUP^b^ ratio	Skimmed milk	Peak lactation	USA	Holstein	Fresh, bronopol and natamycin addition, snap frozen (−80 °C)	Centrifugation, sonication	HPLC	^45^
17	type, processing of diet	corn-grain based diet	Skimmed milk	Mid-lactation	China	Holstein	Fresh	Centrifugation	2D Maldi-TOF/TOF-MS	^50^
18	mastitis	3 h post-challenge by Escherichia coli	Skimmed milk	Mid-lactation	Denmark	Holstein-Friesian	Filtered and snap frozen (−20 °C)	Acidification, centrifugation	iTRAQ labelling, SCX, nano-LC-MS/MS	^48^
19	breed	Holstein-Friesian	Skimmed milk	Mid-lactation	Australia	Holstein-Friesian	Bulk milk and snap frozen (−80 °C)	Centrifugation	Nano LC-ESI-MS/MS	^51^
20	breed	Jersey	Skimmed milk	Mid-lactation	Australia	Jersey	Bulk milk and snap frozen (−80 °C)	Centrifugation	Nano LC-ESI-MS/MS	^51^
21	mastitis	healthy	Skimmed milk	Mid-lactation	Spain	Holstein-Friesian	Snap frozen (−80 °C)	Centrifugation	Maldi-MS/MS	^49^
22	mastitis	healthy	Skimmed milk	Mid-lactation	USA	Holstein	Fresh	Centrifugation	2D Maldi-TOF/TOF-PSD	^47^
23	lactation stage	1 d post-partum	Whey	Colostrum	Denmark	Holstein-Friesian	Snap-frozen (−80 °C)	Centrifugation	2D LC-MS/MS	^41^
24	lactation stage	0 to 5 d post-partum	Whey	Colostrum	China	Holstein	Fresh	Centrifugation	iTRAQ labelling, SCX, LC-MS/MS	^43^
25	lactation stage	calving day	Whey	Colostrum	Belgium	Holstein-Friesian	Snap frozen (−20 °C)	Ultracentrifugation	FASP, dimethyl labelling, LC-LTQ-Orbitrap/MS	^44^
26	mammary gland involution	3 d post drying-off	Whey	Drying-off	New Zealand	Holstein-Friesian	Fresh	Centrifugation	Gel electrophoresis LC-MS/MS	^52^
27	mammary gland involution	8 d post drying-off	Whey	Drying-off	New Zealand	Holstein-Friesian	Fresh	Centrifugation	Gel electrophoresis LC-MS/MS	^52^
28	milk characterization	milk fractionation	Whey	Early lactation	Denmark	Holstein-Friesian	Fresh	Centrifugation	2D LC-MS/MS	^13^
29	lactation stage	9 d post-partum	Whey	Early lactation	Belgium	Holstein-Friesian	Snap frozen (−20 °C)	Ultracentrifugation	FASP, dimethyl labelling, LC-LTQ-Orbitrap/MS	^44^
30	breed	Holstein	Whey	Peak lactation	USA	Holstein	Fresh, bronopol and natamycin addition, snap frozen (−80 °C)	Centrifugation, acidification, ultracentrifugation, major proteins depletion	LC-MS/MS	^46^
31	breed	Jersey	Whey	Peak lactation	USA	Holstein	Fresh, bronopol and natamycin addition, snap frozen (−80 °C)	Centrifugation, acidification, ultracentrifugation, major proteins depletion	LC-MS/MS	^46^
32	diet	low RDP:RUP^b^ ratio	Whey	Peak lactation	USA	Holstein	Fresh, bronopol and natamycin addition, snap frozen (−80 °C)	Centrifugation, acidification, ultracentrifugation, major proteins depletion	LC-MS/MS	^45^
33	diet	high RDP:RUP^b^ ratio	Whey	Peak lactation	USA	Holstein	Fresh, bronopol and natamycin addition, snap frozen (−80 °C)	Centrifugation, acidification, ultracentrifugation, major proteins depletion	LC-MS/MS	^45^
34	mammary gland involution	full lactation	Whey	Mid-lactation	New Zealand	Holstein-Friesian	Fresh	Centrifugation	Gel electrophoresis LC-MS/MS	^52^
35	mastitis	0 h post-challenge by *Streptococcus uberis*	Whey	Mid-lactation	United Kingdom	not reported	Frozen	Centrifugation	LC-MS/MS	^53^

^a^LC-MS/MS: Liquid chromatography coupled with tandem mass spectrometry

iTRAQ: Isobaric tags for relative and absolute quantitation

SCX column: strong cation exchange column

FASP: Filter-aided sample preparation

LTQ-Orbitrap/MS: linear ion trap quadrupole-Orbitrap-mass spectrometry

Maldi-TOF/TOF: matrix-assisted laser desorption/ionization (Maldi) time-of-flight/time-of-flight (TOF/TOF)

PSD: Post-source decay

^b^RDP: dietary rumen degradable protein; RUP: rumen undegradable protein.

Methods for protein identification were FASP, dimethyl labelling, LC-LTQ-Orbitrap/MS^44^, electrophoresis gel LC-MS/MS^52^, iTRAQ labelling, SCX and LC-MS/MS^10,22,39,43,48^, Maldi-(TOF/TOF)-MS detection^42,47,49,50^ and from 1 to 15 repetitions of (nano)LC-MS/MS runs^13,15,17,40,41,45,46,51,53^. Protein identifiers (**ID**) reported in the publications were extracted from tables in Portable Document Format (PDF) or from supplementary data files using Tabula software (www.tabula.technology, Last update February 11, 2017). All collected IDs were aggregated in an atlas of proteins using Excel software (2016). Each protein ID was annotated according to i) milk: fraction, processing, and period of lactation; ii) animal phenotype: breed, health status, and production parameters; and iii) protein identification: number of proteins and proteomic methods used. Because publications report different protein nomenclatures, all protein IDs were changed by the corresponding gene names (**GN**), with the Retrieve/ID Mapping tool of the UniProt database (The UniProt^54^), the Protein Identifier Cross-Reference service^55^ and/or the ProteCONVERT tool of the ProteINSIDE web interface^56^ being used to homogenize data and to generate an atlas of GN.

### Categorization of the milk proteome atlas

For the categorization of the database, four milk fractions were defined based on the protein isolation techniques used in laboratories. The milk fractions were i) skimmed milk, with aggregating proteins isolated by centrifugation under 100 000 g combined with or without casein depletion by acidification (14 datasets); ii) whey, with aggregating proteins isolated by centrifugation over 100 000 g (13 datasets); iii) MFGM, with aggregating proteins isolated from cream milk (4 datasets); and iv) exosomes, with aggregating proteins isolated from skimmed milk with a protocol based on a sucrose gradient^12^ (4 datasets). Five lactation stages were defined according to the DIM, to account for differences in the physiological status and energy balance of the dairy cow. The lactation stages were the i) colostrum period, with aggregating proteins detected in colostrum (samples collected during the first 5 days post-partum; 12 datasets); ii) early lactation (between 6 and 21 DIM, 7 datasets); iii) peak lactation (between 22 and 80 DIM, 6 datasets); iv) mid-lactation corresponding to post-peak lactation (after 81 DIM, but not during the dry-off period, 8 datasets); and v) drying-off (from milk collected at 3 and 8 days after stopping regular milking, 2 datasets). The early lactation stage was defined as 6 to 21 DIM, corresponding to classic physiological NEB^6,57–59^ and excluding the colostrum period^60^.

The categorization of the data from the 20 publications using criteria for the milk fractions and lactation stages produced 35 datasets (Table 2) from different cow breeds, experiments and countries. Among the data, the Holstein-Friesian cows were mainly represented. One-half of studies originated from USA, Australia and Denmark. Finally, we discarded those GN identified more than once in each dataset. Based on the 35 datasets, the atlas aggregated 8841 GN corresponding to proteins.

### Limitations

The first limitation of the computational approach was the use of 20 of the 87 relevant publications on cows available in mid-2018. Indeed, 67 publications were excluded due to a focus on mastitis, a lack of information on the lactation period of the milk collection, incomplete milk fractionation details, insufficient description of animals and husbandry conditions, or absent protein ID. Moreover, the conversion of protein ID into GN led to some data loss, mainly of protein isoforms. Another limitation is that the data reported in the literature depended on the success of the protein identification; thus, a protein could be absent either because it was not detectable, non-identified or absent in the milk sample. Thus, the 59 proteins exclusively detected in early lactation milk may be present at other lactation stages but at very low concentration, and thus they were not identified by proteomics. Finally, the atlas aggregates the proteins that were present because they were identified in milk without regard for their abundance.

### Mining of the milk proteome atlas

From the 8841 GN of the global atlas, we discarded GN identified more than once in each fraction, thereby yielding 7135 unique GN; we did the same for each lactation stage, eventually yielding 6323 GN. The protein lists were compared using Venn diagrams (Draw Venn^61^ tool, VIB / Ugent) to identify proteins specific of a milk fraction (whatever the lactation stage) and of a lactation stage (whatever the milk fraction).

The resulting lists were mined using the ProteINSIDE webservice^56^. Briefly, lists of GN were subjected to a ‘custom analysis’ to access two types of results: (1) the biological knowledge retrieval from bovine (*Bos taurus*) providing mainly the protein’s function, as declared in major databases, tissue-specificity, and subcellular location, and (2) functional annotations according to Gene Ontology (**GO**) by querying the QuickGO database. The ProteINSIDE tool relies mainly on GO enrichment tests (p value_Benjamini and Hochberg < 0.05) among the Biological Process (**BP**), Molecular Function (**MF**) and Cellular Component (**CC**) categories. GO terms imported were selected by evidence codes and agreed on by the curator review in the ProteINSIDE webservice. GN were annotated by GO for bovine but also for human species to benefit from the most complete GO annotation available for human genes. The top 50 enriched GO terms were considered when the significance was lower than P < 0.05.

The potential biomarkers were selected following a workflow with 4 steps. First, we determined the presence/absence of early milk proteins based on the lactation stage. Because of this binary approach (‘all or nothing’ approach), no statistical analysis was applied. Second, we compared the list of the 59 proteins exclusively detected in milk from early lactation with the lists of proteins previously (based on the literature) reported as expressed in key adaptive tissues of lactating cows. Third, we mined each protein using PubMed.gov (NCBI) and the Web of Science (Clarivate Analytics) search engines up to February 2018. Fourth, we performed data mining on protein databases (ProteINSIDE webservice, UniProtKB database) and on pathway webservices (KEGG PATHWAY database, Reactome Pathway database) to complete the analyses with the literature available up to November 2018.

## Supplementary information

Supplementary information
