# Supplementary material for: Milk proteome from in silico data aggregation allows the identification of putative biomarkers of negative energy balance in dairy cows
Source: Sci Rep. 2019 Jul 4;9:9718. doi: 10.1038/s41598-019-46142-7 (PMC6609625; doi:10.1038/s41598-019-46142-7)
Supplement: Supplementary file 1 — Supplementary information [file 41598_2019_46142_MOESM1_ESM.pdf]

Milk proteome from *in silico* data aggregation allows the identification of putative biomarkers of negative energy balance in dairy cows

Mylène Delosière[1]*, José Pires[1], Laurence Bernard[1], Isabelle Cassar-Malek[1], Muriel Bonnet[1]

[1] Université Clermont Auvergne, INRA, VetAgro Sup, UMR Herbivores, F-63122 Saint-Genès-Champanelle, France

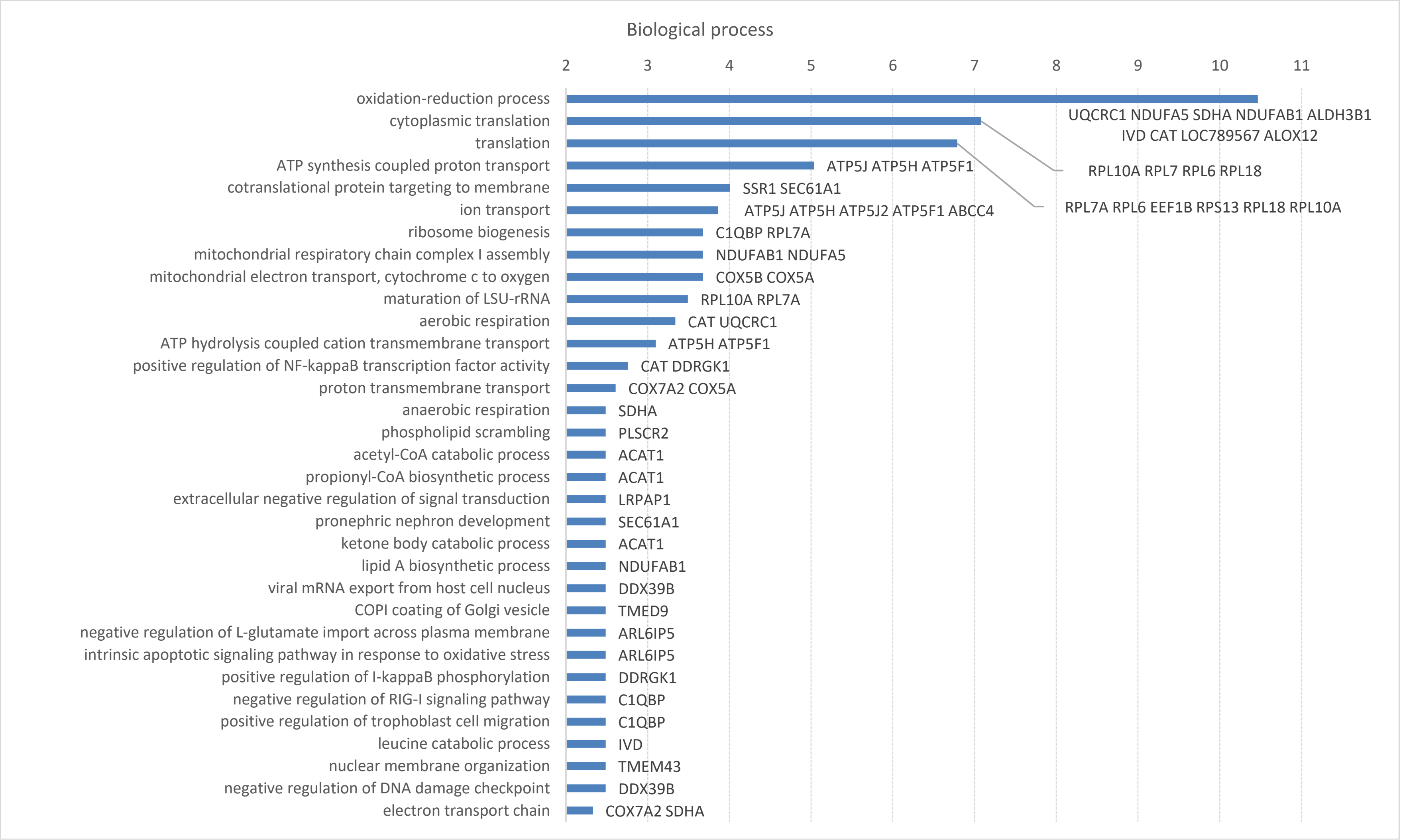

Supplementary Figure 1: The enriched Gene Ontology terms of the Biological Process ontology group annotated 52 proteins among the 59 exclusively detected in early lactation milk. The results were obtained with ProteINSIDE, and enrichments are expressed as –log10 (P value). Log10 (P value) of 3, 2, and 1.3 in the X-axis correspond to a P value of 0.001, 0.01 and 0.05, respectively. P values were corrected by the Benjamini and Hochberg test. The protein gene names annotated by a GO term are listed.

Supplementary Table 1: Lists of the common 95 gene names (GN) from all milk fractions (skimmed, whey, MFM, exosomes) and of the 105 GN from all lactation stages (colostrum, early, peak, mid-lactation and drying-off)

**Common GN present in all fractions**	**Common GN present in all lactation stages**
95	105
ABCG2	A1BG
ACTB	A2M
AHSG	ACTB
ANXA1	AHSG
ANXA2	ALB
APOA1	APOA1
APOA4	APOA4
APOE	APOE
ARHGDIA	AZGP1
AZGP1	B2M
B2M	B4GALT1
B4GALT1	BTN1A1
BTN1A1	C1S
C3	C3
C9	C6
CATHL1	C7
CD14	C9
CD36	CATHL1
CD5L	CATHL2
CD9	CATHL6
CDC42	CD14
CFL1	CD36
CLEC3B	CD5L
CLU	CFB
CRISP3	CFD
CSN1S1	CFH
CSN2	CFI
EEF1A1	CHI3L1
EEF2	CLEC3B
EZR	CLU
F2	CP
FABP3	CREG1
FASN	CRISP3
FGA	CSN1S1
FGB	CSN1S2
FGFBP1	CSN2
FGG	CSN3
FN1	CTSB
FOLR1	DAG1

GC	F2
GDI2	FABP3
GGT1	FGA
GLYCAM1	FGB
GP2	FGFBP1
GRN	FGG
GSN	FN1
HEBP1	FOLR1
HHIPL2	GC
HSP90AA1	GGT1
HSP90AB1	GLYCAM1
HSPA5	GP2
HSPA8	GRN
HSPB1	GSN
IDH1	HP
IGJ	HPX
ITIH4	HSPA5
LBP	IDH1
LPL	IGJ
LPO	ITIH1
LTF	ITIH4
MFGE8	KNG1
MUC1	KNG2
MUC15	KRT1
NPC2	LALBA
NUCB1	LBP
ORM1	LGB
P4HB	LPL
PIGR	LPO
PLIN2	LRG1
PPIA	LTF
PRDX1	MFGE8
RAB18	MUC1
RAB1A	NME2
RAB5C	NPC2
RAB7A	NUCB1
RAP1B	NUCB2
RHOA	ORM1
RNASE4	OS9
S100A8	P4HB
S100A9	PGLYRP1
SAA3	PIGR
SCGB1D	PLG
SELENBP1	PLIN2
SELENOF	PPIA
SERPINA1	PSAP

SLC34A2	PTGDS
TF	QSOX1
TXN	RNASE4
UGP2	SCGB1D
VIM	SDF4
WARS	SERPINA1
XDH	SERPINA3-1
YWHAB	SERPINA3-3
YWHAE	SERPINA3-7
YWHAZ	SERPINA3-8
	SERPINB1
	SERPINC1
	SERPIND1
	SERPINF1
	SERPINF2
	SIL1
	SPP1
	TF
	TTR
	XDH
